# Identification and Mapping of the Clubroot Resistance Gene *CRd* in Chinese Cabbage (*Brassica rapa* ssp. *pekinensis*)

**DOI:** 10.3389/fpls.2018.00653

**Published:** 2018-05-18

**Authors:** Wenxing Pang, Pengyu Fu, Xiaonan Li, Zongxiang Zhan, Sha Yu, Zhongyun Piao

**Affiliations:** Molecular Biology of Vegetable Laboratory, College of Horticulture, Shenyang Agricultural University, Shenyang, China

**Keywords:** Chinese cabbage, *Plasmodiophora brassicae*, clubroot disease, bulked segregant analysis, pathogen resistance gene, CR cultivars

## Abstract

The rapid spread of clubroot disease, which is caused by *Plasmodiophora brassicae*, threatens Brassicaceae crop production worldwide. Breeding plants that have broad-spectrum disease resistance is one of the best ways to prevent clubroot. In the present study, eight Chinese cabbage germplasms were screened using published clubroot-resistant (CR) loci-/gene-linked markers. A CR gene Crr3 potential carrier “85-74” was detected which linked to marker BRSTS61; however, “85-74” shows different responses to local pathogens “LAB-19,” “LNND-2,” and “LAB-10” from “CR-73” which harbors Crr3. We used a next-generation sequencing-based bulked segregant analysis approach combined with genetic mapping to detect CR genes in an F_2_ segregant population generated from a cross between the Chinese cabbage inbred lines “85-74” (CR) and “BJN3-1” (clubroot susceptible). The “85-74” line showed resistance to a local pathogen “LAB-19” which was identified as race 4; a genetic analysis revealed that the resistance was conferred by a single dominant gene. The CR gene which we named *CRd* was mapped to a 60 kb (1 cM) region between markers yau389 and yau376 on chromosome A03. *CRd* is located upstream of *Crr3* which was confirmed based on the physical positions of *Crr3* linked markers. The identification of *CRd* linked markers can be applied to marker-assisted selection in the breeding of new CR cultivars of Chinese cabbage and other *Brassica* crops.

## Introduction

Chinese cabbage (*Brassica rapa* ssp. *pekinensis*) is one of the most important leafy head vegetables cultivated in China, Korea, and Japan. Its production has been undermined by the rapid spread of clubroot disease, resulting in major economic losses. The soil-borne obligate plant pathogen *Plasmodiophora brassicae* Woronin causes clubroot in *Brassica* crops, blocking nutrient and water transport (Voorrips et al., 2003). The life cycle of *P. brassicae* has not been well understood until now. The *P. brassicae* infection starts from primary zoospores releasing and causes root hair infection and then primary zoospore or secondary zoospores induce cortical infection leading to the formation of galls on the roots (McDonald et al., 2014). Crop rotation and application of fluazinam and cyazofamid can effectively reduce the viability of resting *P. brassicae* spores and prevent infection (Ransom et al., 1991; Suzuki et al., 1995; Wallenhammar, 1996; Mitani et al., 2003; Townley and Fox, 2003; Miller et al., 2007; Kutcher et al., 2013; Peng et al., 2014). Although these approaches alleviate the symptoms of clubroot, they do not eradicate the disease. Breeding of clubroot-resistant (CR) cultivars is a desirable strategy for controlling clubroot owing to its advantages such as low cost and environment friendliness.

*CRa*, *CRb*, *CRc*, *CRk*, *Crr1*, *Crr2*, *Crr3*, *Crr4*, *PbBa3.1*, *PbBa3.3*, and *QS_B3.1* genes have been identified in European fodder turnips (Matsumoto et al., 1998; Suwabe et al., 2003, 2006; Hirai et al., 2004; Piao et al., 2004; Sakamoto et al., 2008; Chen et al., 2013; Pang et al., 2014). Most of these genes are from different genetic resources and are associated with distinct *P. brassicae* pathotypes. *CRa* and *CRb* were resistant to race 2 and race 4, respectively. *Crr1*, *Crr2*, and *Crr4* from Siloga were resistant to Ano-01 and Wakayama-01. *CRc* and *CRk* from Debra were resistant to isolates M85 and K04. *Crr3* was detected from Milan White which was resistant to isolate Ano-01. *PbBa3.1* and *PbBa3.3* were detected from ECD04 conferring resistance to Pb2 and Pb7, respectively. Several CR genes were also recently mapped in *B. rapa* (Yu et al., 2016; Huang et al., 2017). Some of the genes and loci including *CRa*, *CRb*, and *QS_B3.1*, *Crr3*, *CRk*, and *PbBa3.3* were clustered in a proximal region of chromosome A03 in *B. rapa*; whether they represent a single or multiple genes remains to be determined. *CRa* and *Crr1a* have been cloned and are known to encode Toll-interleukin-1 receptor-like domain-nucleotide binding site-leucine-rich repeat (TIR-NBS-LRR) proteins. It was reported that *CRb* is the same as *CRa* (Hatakeyama et al., 2017); however, the identities of the remaining CR genes require confirmation.

Molecular markers linked to CR loci or genes are essential for pyramiding several CR genes into one cultivar through marker-assisted selection (MAS). MAS has been successfully used for transforming CR genes into Chinese cabbage (Yoshikawa, 1981; Zhang et al., 2012). However, *B. rapa*, *Brassica oleracea*, and *Brassica napus* plants known to harbor genes conferring specific resistance to *P. brassicae* (Rocherieux et al., 2004; Werner et al., 2008; Chen et al., 2013) have all exhibited loss of resistance within a few years (Tjallingii, 1965; Kuginuki et al., 1999). Therefore, identifying novel CR genes or alleles associated with resistance to different pathotypes is essential for overcoming the challenges of co-existing pathotypes and the rapid mutation rate of *P. brassicae* in the field.

Next-generation sequencing (NGS)-based bulked segregant analysis (BSA) is a powerful tool for mapping disease resistance gene/genes that has been applied to *Arabidopsis*, rice (*Oryza sativa*), sorghum (*Sorghum bicolor*), soybean (*Gycin emax*), wheat (*Triticum turgidum*), and cotton (*Gossypium*; Trick et al., 2012; Yang et al., 2013; Uchida et al., 2014; Han et al., 2015; Song et al., 2017; Zhu et al., 2017). In the present study, we found the Chinese cabbage inbred line “85-74,” which exhibited resistance to the local pathogen “LAB-19” (race 4) and was distinct from CR germplasms harboring *CRa*, *Crr1*, and *Crr3*. We employed NGS-based BSA to identify the CR gene/genes in “85-74.” Our results can provide a basis for breeding new CR cultivars of Chinese cabbage.

## Materials and Methods

### Plant Materials

Eight Chinese cabbage germplasms including “CR Shinki,” “CR-77,” “CR-20,” “CR-75,” “CR-26,” “CR-73,” “85-74,” and “BJN3-1” were used in this study. “CR Shinki” harbors the *CRa* and *CRb* gene, and “CR-77,” “CR-20,” “CR-75,” “CR-26,” “CR-73,” and “85-74” were genotyped using published CR loci-/gene-linked markers (Saito et al., 2006; Suwabe et al., 2006; Sakamoto et al., 2008; Ueno et al., 2012; Zhang et al., 2012; Chen et al., 2013; Hatakeyama et al., 2013; Pang et al., 2014). To evaluate the resistance of CR resources against different pathotypes of *P. brassicae*, eight CR inbred lines were inoculated with 11 local pathogens. Their pathotypes were classified based on Williams’ clubroot differential set (Williams, 1966), which includes Jersey Queen, Badger shipper, Laurentian, and Wilhelmsburger. The clubroot-susceptible inbred line of Chinese cabbage “BJN3-1” served as a negative control.

An analysis of CR loci-/gene-linked markers revealed that “85-74” and “CR-73” harbored the *Crr3* gene, but showed distinct responses to three local pathogen isolates. We therefore crossed “85-74” and “BJN3-1” and then self-pollinated the offspring to produce an F_2_ population; 432 F_2_ individuals were used for genetic analysis and 127 were self-pollinated to generate an F_3_ population that was used for CR tests.

### Pathogen Inoculation and CR Tests

A total of 11 field isolates were collected from infected Chinese cabbage or canola from different province of China and maintained on the roots of a susceptible Chinese cabbage “91-12” and stored in -20°C until required. These pathogens were classified according to Williams’ clubroot differential set and used to evaluate the resistance of eight Chinese cabbage germplasms; additionally, 24 seeds from each of eight CR Chinese cabbage inbred lines along with Williams’s differential set were grown for pathogen screening in the spring of 2014.

A total of 36 seeds of F_1_ individuals and 432 seeds of F_2_ individuals of “85-74” and “BJN3-1” were grown and inoculated with “LAB-19” in 2015; 36 seeds from each of 127 F_3_ populations were grown for the “LAB-19” inoculation test in 2016. All plants were grown in 72-well multipots and maintained in a greenhouse at 20–25°C under a 16-h photoperiod. Resting spores were prepared and inoculation was performed as previously described (Pang et al., 2014). For inoculation, 1 ml of spore suspension was applied to the bottom of the stem base of each 7-days-old seedling; disease resistance was evaluated 6 weeks later.

### DNA Extraction and Pool Construction

Young leaves from eight Chinese cabbage germplasm were collected and used for characterization of known CR loci/genes. Young leaves from 127 F_2_ individuals were sampled and used for genome sequencing and gene mapping. The genomic DNA was extracted according to the cetyl trimethylammonium bromide method (Li et al., 2010), with minor modifications. DNA concentration was determined with a NanoDrop 2000 spectrophotometer (Thermo Fisher Scientific, Waltham, MA, United States). Highly resistant and susceptible F_3_ families were selected and equal amounts of DNA from selected F_2_ individuals were mixed together to form resistant and susceptible pools (R- and S-pool, respectively) that were sequenced along with “85-74” and “BJN3-1” with the HiSeq 2500 system (Illumina, San Diego, CA, United States).

### Analysis of Markers Linked to Previously Identified CR Genes

Chinese cabbage germplasms were genotyped for known CR loci/genes identification using linked markers (Saito et al., 2006; Suwabe et al., 2006; Sakamoto et al., 2008; Ueno et al., 2012; Chen et al., 2013; Hatakeyama et al., 2013; Pang et al., 2014; Zhang et al., 2014). DNA fragments were amplified by PCR and the susceptible “BJN3-1” line was used as a control. PCR products were separated on a standard agarose (2%) or polyacrylamide (6%) gel and their sizes were compared to that of known resistance genes.

### Sequencing and Bioinformatics Analysis

Genome sequencing of the two parental lines and R- and S-pools and statistical analysis of bioinformatics data were carried out by Annoroad Co.^1^ A library of 300–500-bp insert size was constructed and paired-end sequenced on an Illumina HiSeq 2500 platform. Raw data were processed with Perl scripts to ensure data quality for subsequent analyses. The adopted filtering criteria are as follows: (1) remove the adaptor-polluted reads (reads containing more than five adapter-polluted bases were regarded as adaptor-polluted reads and would be filtered out); (2) remove the low-quality reads; reads with the number of low-quality bases (phred Quality value less than 19) accounting for more than 50% of total bases are regarded as low-quality reads; and (3) remove reads with number of *N* bases accounting for more than 5%. As for paired-end sequencing data, both reads would be filtered out if any read of the paired-end reads are adaptor-polluted. The obtained Clean Data after filtering will be carried out on statistics analyses on its quantity and quality, including Q30, data quantity, base content statistics, etc. Clean reads were aligned to the reference genome sequence “Chiifu-401-42” from the Ensemble Genome database^2^ using Burrows–Wheeler Aligner v.0.7.12 (Li and Durbin, 2009). Samtools v.1.2 (Li et al., 2009) was used to sort the reads, and duplicate reads obtained by PCR were removed using the MarkDuplicates command of Picard tools v.1.13^3^. Reads mapped to two or more sites were filtered out. Statistical analyses were carried out using an in-house Perl script.

The Genome Analysis Toolkit (GATK; McKenna et al., 2010) HaplotypeCaller function was used for single nucleotide polymorphism (SNP) and insertion-deletion (InDel) calling. The SNPs and InDels were filtered with the GATK VariantFiltration protocol before further analysis with the following settings: QD < 2.0, MQ < 40, DP < 4, MQRankSum < -12.5. Annotation was performed using ANNOVAR (Wang et al., 2010) for all qualified variants based on the GFF file.

### BSA Mapping Using Sequencing Data

To detect candidate loci associated with CR, the SNP index was calculated for all variants (Takagi et al., 2013). To reduce the impact of sequencing and alignment errors, we filtered out variations that met any of the following conditions: (1) loci in parents were heterozygous; (2) depths of variation were <10 or positions were not covered in parents or bulks; (3) variations in SNP index in both bulks were <0.3 or >0.7; or (4) variations were not on chromosomes (e.g., they were on a scaffold). All remaining variants were retained for further analysis. We slid along the genome with a 1-Mb window at a step size of 100 kb to calculate the mean SNP index, and subtracted the SNP index value of the R-pool from that of the S-pool to obtain the ΔSNP index (Takagi et al., 2013). Confidence intervals at 0.1, 0.5, and 0.01 levels were determined by computer simulation; the threshold was set at a 0.01 confidence level to identify candidate quantitative trait loci. The SNPs and InDels in the confidence region were selected and validated through Sanger sequencing.

### *CRd* Mapping and Candidate Genes Analysis

The reference genome sequence from the Ensemble Genome database was downloaded and used for marker development in the *CRd* candidate region. Simple sequence repeat (SSR) markers were developed using SSR Hunter v.1.3 (Li and Wan, 2005). The two parental lines along with R- and S-pools were used to develop markers linked to the CR gene. A genetic map was constructed with the developed and previously published markers using JoinMap v.4.0 (Stam, 1993; Van Ooijen, 2006). *CRd* closely linked markers were validated in natural population. The candidate genes in the *CRd* region were compared with 244 resistance genes in *B. rapa*^4^.

### Semi-Quantitative RT-PCR Analysis

Total RNA was isolated from 0 day, 7 days, 10, and 13 days after inoculation (DAI) of *P. brassicae* of “85-74” and “BJN3-1” root tissue using an Easy-BLUETM Total RNA Extraction Kit (Invitrogen, United States). The total RNA from each plant sample amounting to 5 μg was combined with random hexamer primers in a Super Script first-strand cDNA synthesis system according to the manufacturer’s instructions (Invitrogen, United States). Complementary DNA was diluted 10-fold, and 1 μl of the diluted cDNA was used in each 20 μl PCR mixture. Sequence information from *B. rapa* was used for RT-PCR primers design. Standard PCR was performed, with 5 min denaturation at 94°C followed by 25 cycles of 94°C for 30 s, 55°C for 30 s, and 72°C for 60 s. The PCR products were analyzed following electrophoresis on a 1% agarose gel.

## Results

### Characterization of Chinese Cabbage Germplasm

To characterize CR resources of Chinese cabbage, eight inbred lines of CR Chinese cabbage were genotyped with known CR gene linked markers and infected with 11 different local isolates of *P. brassicae*. “CR-77” and “CR-20” were found to carry *Crr1* and *CRa*; “CR-75” and “CR Shinki” harbored *CRa* and *CRb*; “CR-73” harbored *CRk* and *Crr3*. “85-74” was a potential carrier of *Crr3*, which was linked to BRSTS61. “BJN3-1” had no CR loci, and “CR-26” carried unknown CR gene/genes (**Table 1**). Based on Williams’ classification system, local pathogens “AHXC-68,” “LAB-16,” “LNXM-1,” “AHHS-62,” “LAB-19,” and “LNND-2” were identified as pathotype 4; “LAB-7” and “LAB-10” were pathotype 2; “HBLC-31” and “AHHS-65” were pathotype 7; and “AHHS-65” was pathotype 11. CR inbred lines showed variable resistance depending on the presence of infection with these pathogens. The negative control “BJN3-1” was infected by all pathogens. “CR-77” and “CR-20” showed resistance to all 11 pathogens; “85-74” was susceptible to “LNND-2” and “LAB-10,” but resistant to the other pathogens tested in this study; “CR Shinki” was susceptible to “LNXM-1” and “AHHS-62,” but resistance to the other pathogens; “CR-75” and “CR-26” were susceptible to “LNXM-1” and “AHHS-62,” respectively; and “CR-73” was susceptible to “LAB-19” (**Table 1**). “CR-73” and “85-74” showed distinct resistance responses to “LAB-19,” “LNND-2,” and “LAB-10.” This suggests that “85-74” and “CR-73” harbor different CR genes or alleles. Based on these results, “LAB-19” was selected to phenotype F_2_ and F_3_ populations derived from the “85-74” and “BJN3-1” cross.

**Table 1:** Clubroot disease resistance test of Chinese cabbage germplasms and pathotypes identification using Williams’ clubroot differential set.

Materials	CR gene/genes	Local pathogens										
		AHXC-68	LAB-16	LNXM-1	AHHS-62	LAB-19	LNND-2	LAB-10	LAB-7	HBLC-31	AHHS-65	HBSY-32
CR Shinki	*Cra*, *CRb*	-	-	+	+	-	-	-	-	-	-	-
CR-75	*Cra*, *CRb*	-	-	+	-	-	-	-	-	-	-	-
CR-26	unknown	-	-	-	+	-	-	-	-	-	-	-
CR-73	*CRk*, *Crr3*	-	-	-	-	+	-	-	-	-	-	-
85-74	*CRd*	-	-	-	-	-	+	+	-	-	-	-
CR-77	*Crr1*, *Cra*	-	-	-	-	-	-	-	-	-	-	-
CR-20	*Crr1*, *Cra*	-	-	-	-	-	-	-	-	-	-	-
BJN3-1	None	+	+	+	+	+	+	+	+	+	+	+
Jersey Queen		+	+	+	+	+	+	+	+	+	+	-
Badger shipper		+	+	+	+	+	+	+	+	+	+	+
Laurentian		+	+	+	+	+	+	+	+	-	-	+
Wilhelmsburger		+	+	+	+	+	+	-	-	-	-	+

*+ represents susceptible reactions and - represents resistant reactions.*

### Phenotype Evaluation and R- and S-Pool Construction

To investigate the inheritance of the resistance to local pathogen “LAB-19,” parental lines, F_1_, and 432 F_2_ individuals were inoculated with 1 × 10^7^ spores⋅ml^-1^. The “85-74” and F_1_ lines were highly resistant whereas “BJN3-1” was susceptible. Among 432 F_2_ individuals, 321 and 106 were resistant and susceptible, respectively, and exhibited a 3:1 segregation ratio at a 0.05 level of probability (**Table 2**). These results indicated that CR is controlled by a single dominant gene in “85-74.” Parental lines with 127 F_3_ families were then inoculated with “LAB-19” in 2016. R- and S-pools were constructed by selecting 19 highly CR and 16 susceptible F_2_ individuals depending on the F_3_ family phenotype.

**Table 2:** Genetic analysis of clubroot resistance in the F_2_ population.

Materials	Resistant plants	Susceptible plants	χ^2^	χ^2^ 0.05
“85-74”	30	0		
“BJN3-1”	0	29		
F_1_ population	30	0		
F_2_ population	321	106	0.007	3.841

### Sequencing Data Analysis

Sequencing data were generated with Illumina HiSeq 2500 with an average insert size of about 350 bp. A total of 250,343,998, 249,577,324, 250,291,386, and 249,507,094 raw reads were obtained from “85-74,” “BJN3-1,” and R- and S-pools (SRA accession: SRP136862), respectively. Clean data were obtained after removing adapter-polluted and low-quality reads and unknown bases (*N* > 5%), yielding minimum and maximum clean Q30 base rates of 90% and 92.34%, respectively (**Table 3**). Genome coverage ranged from 90.24 to 91.63%, and average depths were 86.26×, 84.22×, 83.49×, and 85.34× for “85-74,” “BJN3-1,” and R- and S-pools, respectively.

**Table 3:** Quality control of sequencing data.

Sample	“85-74”	“BJN3-1”	R-pool	S-pool
Raw reads	250,343,998	249,577,324	250,291,386	249,507,094
Raw bases	37,551,599,700	37,436,598,600	37,543,707,900	37,426,064,100
Clean reads	243,624,928	243,358,774	244,980,058	243,367,696
Clean bases	36,543,739,200	36,503,816,100	36,747,008,700	36,505,154,400
Clean read rate (%)	97.32	97.51	97.88	97.54
Low-quality reads	4,481,482	4,157,506	3,183,920	3,906,490
Low-quality read rate (%)	1.79	1.67	1.27	1.57
Ns reads	68,718	70,224	1,542	70,616
Ns read rate (%)	0.03	0.03	0	0.03
Adapter-polluted reads	2,168,870	1,990,820	2,125,866	2,162,292
Adapter-polluted read rate (%)	0.87	0.8	0.85	0.87
Raw Q30 base rate (%)	89.09	89.46	91.68	89.9
Clean Q30 base rate (%)	90	90.3	92.34	90.71

**FIGURE 1:**
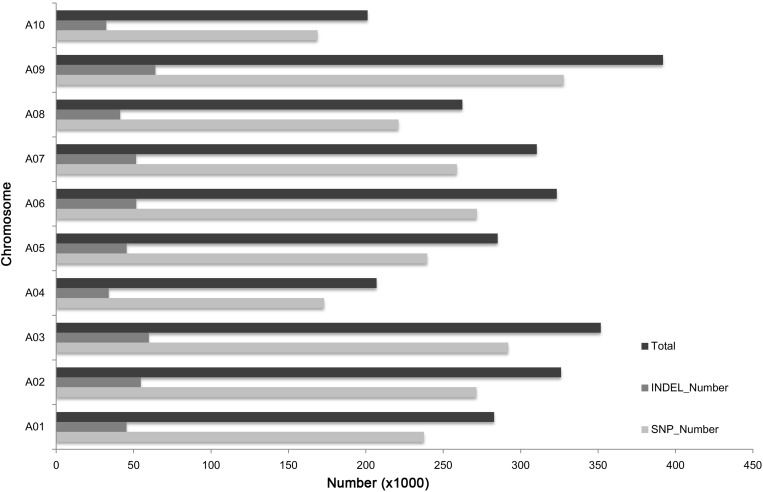
Distribution of SNPs and InDels on each chromosome.

In total, 2,941,775 SNPs and InDels were detected between “85-74” and “BJN3-1” (**Figure 1**). The average number of sequence variations on the 10 chromosomes was 294,177, with chromosomes A09 and A10 having the highest and lowest number of variations, respectively. Chromosome A03 had a comparatively high number and density of variations in a specific region.

### Association Analysis

**FIGURE 2:**
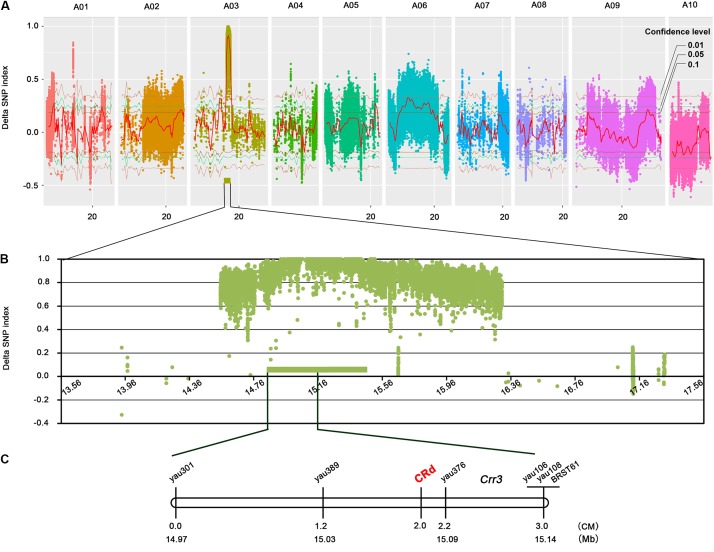
*CRd* mapping by NGS-based BSA and genetic mapping approaches. **(A)** Genome-wide ΔSNP index Manhattan plots and marker-trait association with 0.1, 0.05, and 0.01 confidence levels. **(B)** ΔSNP index Manhattan plots in candidate region; the green rectangle indicates the core region. **(C)** Genetic/physical map of the region harboring *CRd* on chromosome 3.

To calculate SNP index, we filtered out sequence variations that met the above-described conditions. Of the 2,941,775 variations, 599,797 were to calculate SNP index (**Figure 2A**). According to the ΔSNP index value, a 3.94-Mb candidate region from 13.57 to 17.51 Mb was identified on chromosome A03 at a 0.01 confidence level (**Figure 2B**). A total of 19,664 SNPs and 4450 InDels were found in the candidate region; 1953 out of 5861 variations in exons caused changes of amino acid sequence (**Supplementary Table S1**). Twenty pairs of primers were designed for InDels validation (**Supplementary Table S2**). Seven pairs of primers were not amplified PCR production and the rest of 13 primers produced single band. The PCR products were sequenced and shown exactly same with our BSA-sequencing data (**Supplementary Figure S1**). The candidate genes in the *CRd* region were compared with 244 resistance genes in *B. rapa*^5^. Four resistance genes were identified which encode TIR-NBS-LRR protein, including Bra001160, Bra001161, Bra001162, and Bra001175 with 20, 4, 42, and 81 sequence variations, respectively, in the exons.

### *CRd* Mapping and Candidate Genes Analysis

A core region of 577 kb was found on chromosome A03 with an extremely high average ΔSNP index of 0.9631 (**Figure 2B**). We designed eight SSR primer pairs within this region and screened for polymorphisms between the two parent lines. Five polymorphic markers were identified including yau301, yau389, yau376, yau106, and yau108; these were used to genotype 127 F_2_ individuals (**Figure 2C** and **Supplementary Table S3**). The *Crr3* linked marker BRSTS61 showing polymorphism between “85-74” and “BJN3-1” was used to compare the mapping locations of *CRd* and *Crr3*.

A genetic map of the region surrounding the *CRd* gene was constructed based on the genotypes of seven markers. *CRd* was mapped to a 1 cM region with the flanking markers yau389 and yau376 (**Figure 2C**). Alignment of marker sequences to the reference genome sequence of *B. rapa* revealed a physical distance between yau389 and yau376 of about 60 kb. *CRd* is located upstream of *Crr3* was confirmed based on the physical position of *Crr3* linked markers. *CRd* closely linked markers yau389 and yau376 were validated in natural population and the cultivars which harbor *CRd* gene all showed resistant to isolate “LAB-19” (**Supplementary Figure S2** and **Supplementary Table S4**). Total four genes Bra001160, Bra001161, Bra001162, and Bra001175 which encode TIR-NBS-LRR protein were identified in the *CRd* candidate region.

### Semi-Quantitative RT-PCR Analysis

**FIGURE 3:**
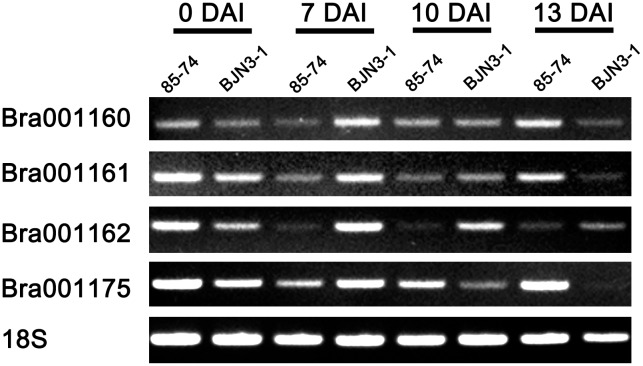
Expression levels of Bra001160, Bra001161, Bra001162, and Bra001175 genes in roots of “85-74” and “BJN3-1.”

Total four genes Bra001160, Bra001161, Bra001162, and Bra001175 which encode TIR-NBS-LRR protein were identified in the *CRd* candidate region. To examine the expression characteristics of these four genes from “85-74” and “BJN3-1,” we performed RT-PCR analysis with a common primer set (18S) and resistance gene-specific primer (**Supplementary Table S5**). As shown in **Figure 3**, 18S expressed in “85-74” and “BJN3-1” from 0 DAI to 13 DAI. Bra001160, Bra001161, and Bra001175 were more highly expressed in “85-74” at 13 DAI. Bra001162 was more highly expressed in “BJN3-1” at 7 DAI, 10 DAI, and 13 DAI.

## Discussion

In this study, we used an NGS-based BSA strategy to map the novel CR gene *CRd* in an F_2_ population of *B. rapa*. Previously identified CR loci or genes in *B. rapa* have been associated with resistance to specific pathotypes of *P. brassicae*. For instance, the *CRa* gene confers resistance to *P. brassicae* isolate M85, which belongs to race 2 according to Williams’s classification (Williams, 1966; Ueno et al., 2012), whereas *CRb* (Piao et al., 2004) and *Crr1* (Hatakeyama et al., 2013) confer resistance to race 4 and Ano-01 (an unknown pathotype), respectively. In the present study, “85-74” showed resistance to all pathotypes of race 2, 4, 7, and 11, except for “LAB-10” (race 2) and “LNND-2” (race 4). “CR-73” showed resistance to all tested isolates except to “LAB-19” (race 4). The variable responses to “LAB-19,” “LAB-10,” and “LNND-2” indicate that “85-74” and “CR-73” have distinct genetic backgrounds. Moreover, “CR-75” was identified carrying *CRa* and *CRb* using published CR loci-/gene linked markers and “CR Shinki” harbored *CRa* and *CRb* also. However, “CR-75” showed resistant to “AHHS-62” while “CR Shinki” not which indicates that “CR-75” is highly possible carrying other unknown CR gene/genes need to explore through genetic mapping. A given pathotype identified according to Williams’s classification system is expected to produce the same response in hosts; however, pathotype 4 had different infectivity in the Chinese cabbage germplasm inoculation tests. “LAB-7” and “LAB-10” were identified as pathotype 2; however, only the latter infected “85-74” successfully. These results indicate that the Williams’s classification system has a limited capacity for distinguishing co-existing isolates.

Clubroot disease is threating all of the brassica crops. To reduce the economic losses, CR genes have been investigated in canola (*Rcr1* and *Rcr4*, Yu et al., 2016, 2017), cabbage (*Anju1*, *Anju2*, *Anju4*, and *GC1*, Tomita et al., 2013), and Chinese cabbage (*CRa*, *Crr1*, and *CRb*, Ueno et al., 2012; Hatakeyama et al., 2013; Zhang et al., 2014.) These CR genes and their closely linked markers exploration in brassica crops have been greatly improved the CR breading through MAS strategy. Breeding of CR cultivars is the most environment friendliness and economically effective strategy for controlling clubroot. Plant disease resistance genes are abundant and are clustered together in the genome (Michelmore and Meyers, 1998; Wang et al., 2011). Most of the CR loci or resistance genes reported to date in *B. rapa* are clustered on chromosome A03 in two specific genomic regions (*CRa*, *CRb*, and *QS_B3.1* at one locus and *CRk* and *Crr3* at another). *CRd* is in the same genomic region as *CRk* and *Crr3*. The *CRk* linked marker HC688 was not polymorphic in our population. The sequences of the *Crr3* linked markers BrSTS78 and BrSTS33 were searched in the Ensemble Genome database to further distinguish between *CRd* and *Crr3*, and were found to be located at 15.091 and 15.331 Mb, respectively, on chromosome A03. Meanwhile, *CRd* was mapped to between yau389 (15.029 Mb) and yau376 (15.089 Mb). These results confirm that *CRd* is located upstream of *Crr3*. Moreover, “85-74” and “CR-73” (harbor Crr3 resistant gene) showed different responses to “LAB-19,” “LAB-10,” and “LNND-2.” Thus, *CRd* is possible to be a novel CR gene distinct from those previously identified on chromosome A03 of *B. rapa*.

Most of the disease resistance genes encoding NBS-LRR proteins confer pathogen race-specific resistance (Flor, 1956; Meyers et al., 2003). It was previously reported that *CRa* and *Crr1* encode TIR-NBS-LRR protein. In the present study, Bra001162 was more highly expressed in “BJN3-1” at 7 DAI, 10 DAI, and 13 DAI indicated that Bra001162 may not associate with CR, while Bra001160, Bra001161, and Bra001175 were more highly expressed in “85-74” than “BJN3-1” at 13 DAI (**Figure 3**). Therefore, Bra001160, Bra001161, and Bra001175 are highly possible to be candidate genes of *CRd*. These results will be helpful for the *CRd* gene cloning and validation of transgenic lines in future study.

*CRd* was mapped into a 1 cM region on chromosome A03 of the *B. rapa* genome in a small F_2_ segregant population. However, it was found to be anchored to a relatively short 60 kb region based on the reference genome of *B. rapa*, indicating the presence of a recombination hotspot at this location. Previously studies have shown that such hotspots in *Arabidopsis thaliana* are accession-specific or vary depending on the cross (Drouaud et al., 2006, 2007; Kim et al., 2007; Salomé et al., 2012). It is also possible that “85-74” harbors a large insertion that is not present in the reference genome.

## Conclusion

We identified the *CRd* gene in a CR population of *B. rapa*. Our findings may be useful for breeding cultivars of Chinese cabbage and other Brassica crops with broad-spectrum resistance to multiple *P. brassicae* pathotypes.

## Author Contributions

WP analyzed the data and drafted the manuscript. PF performed the experiments and data analysis. XL and ZZ helped in the data analysis and experiments. ZP conceived the study, participated in its coordination, and helped to draft the manuscript. All authors have read and approved the final manuscript.

## Conflict of Interest Statement

The authors declare that the research was conducted in the absence of any commercial or financial relationships that could be construed as a potential conflict of interest.

## Footnotes

1 http://www.annoroad.com/

2 ftp://ftp.ensemblgenomes.org/pub/release-32/plants/fasta/brassica_rapa/dna/Brassica_rapa.IVFCAASv1.dna.toplevel.fa.gz

3 http://broadinstitute.github.io/picard/

4 http://brassicadb.org/brad/

5 http://brassicadb.org/brad/

## Supplementary Material

FIGURE S1 Agarose gel of PCR product of InDels validation primer set.

FIGURE S2 Validation of *CRd* closely linked marker **(A)** yau376 and **(B)** yau389 using natural population. Lanes 1–25 are materials that have been listed in Supplementary Table S4. Black arrow indicates the clubroot-resistant band.

TABLE S1 Details of SNP index and annotation for 1953 SNPs and InDels that can cause changes in the amino acid sequence of the candidate region.

TABLE S2 Primers designed for InDels validation and PCR product sequences.

TABLE S3 Markers used for genetic map construction in this study.

TABLE S4 Materials used for natural validation in this study.

TABLE S5 Primer set used for semi-quantitative RT-PCR analysis.
