# Supplementary figures and images for: Identification and Mapping of the Clubroot Resistance Gene CRd in Chinese Cabbage (Brassica rapa ssp. pekinensis)
Source: Front Plant Sci. 2018 May 18;9:653. doi: 10.3389/fpls.2018.00653 (PMC5968122; doi:10.3389/fpls.2018.00653)

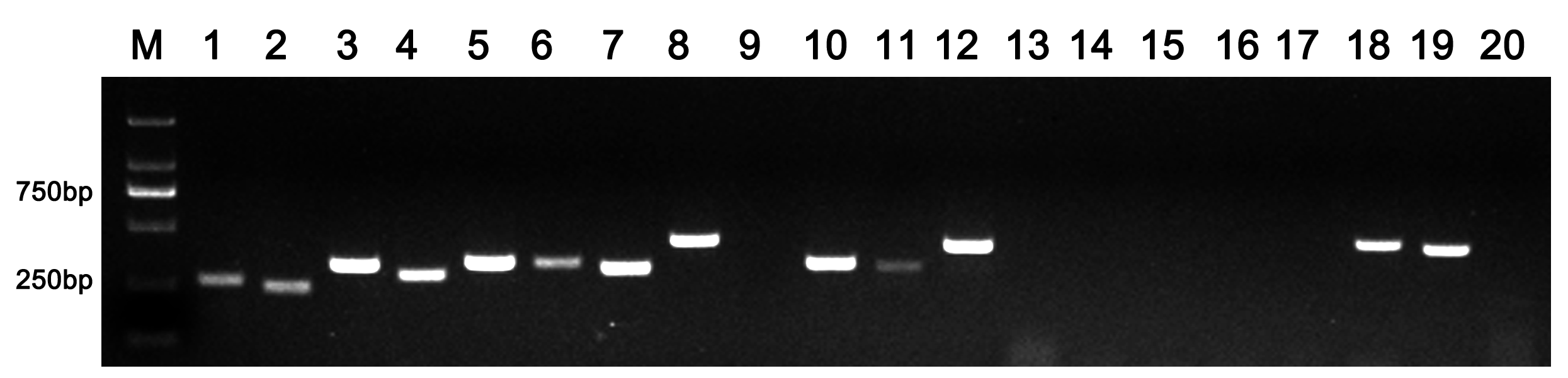

Supplement: FIGURE S1 — Agarose gel of PCR product of InDels validation primer set. [file Image_1.TIF]

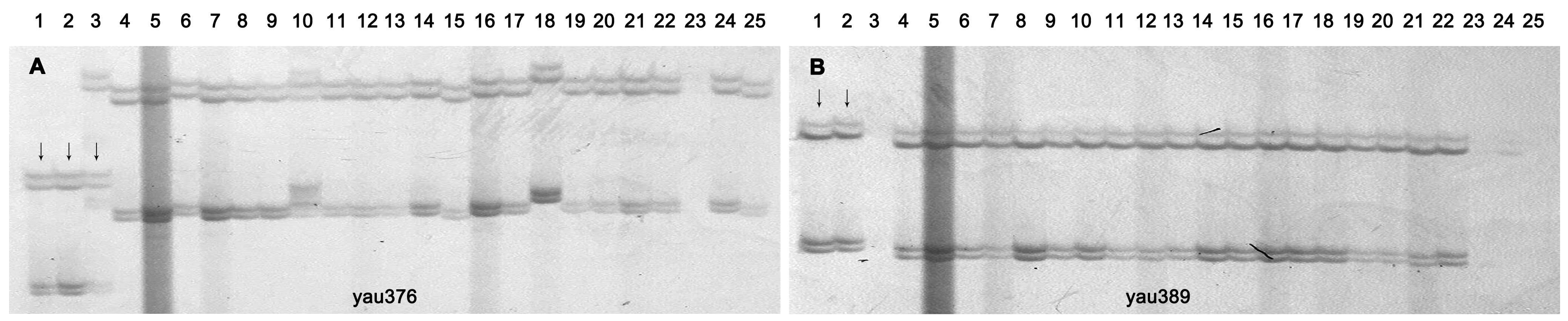

Supplement: FIGURE S2 — Validation of CRd closely linked marker (A) yau376 and (B) yau389 using natural population. Lanes 1–25 are materials that have been listed in Supplementary Table S4. Black arrow indicates the clubroot-resistant band. [file Image_2.TIF]
